# The Story of the Insane from Year to Year

**Published:** 1891-08-08

**Authors:** 


### The Story of the Insane from Year to Year.

(Fourth Series.)

[Annual Reports, Pamphlets, &c., should be addressed to The Editor, The Lodge, Porchester Square, London, W.]

We have always held that the highest number of patients congregated under one asylum roof and one management should be 1,000, and we believe 700 or 800 would be a much better limit. It is surely the duty of everyone whose voice can be heard at all to protest against the erection of these gigantic asylums, which can only be looked upon as gigantic mistakes. It is not only that old asylums have been added to until their proportions are unwieldy, and their proper management difficult or impossible, but new buildings have been put up for absurd numbers. At present one of these mammoths is being built at Claybury for, it is said, 2,000 patients. All asylum plans have to obtain the approval of the Home Secretary. We would ask whether some means could not be found whereby the matter could be brought under notice of the Home Office, and the repetition of these mistakes rendered impossible for the future.

### MIDDLESEX COUNTY ASYLUM AT WANDSWORTH.

At the close of 1890 the number of patients in this asylum had risen to 1,089, showing an increase of eleven during the year. Ninety patients were discharged recovered, being at the rate of 35·8 per cent. of the admissions. Seventy-four of the patients died. Calculated on the average number resident, this number gives a percentage of 6·8, which must be looked on as a low death rate. Brain diseases account for 24 of the deaths, and phthisis for 10. As to the causes of insanity, we find drink and heredity, as usual, leading the way, the former with 33, and the latter with 63. When speaking of restraint, Mr. Gardiner Hill says: "Because this form of treatment is unpopular it is no reason why it should be discontinued altogether, for there are exceptional cases in which the use of restraint may be fully justified." Coming from the son of the man who introduced our modern non-restraint system this view ought to carry much weight; and we would add that it (restraint) is at times not only "fully justified," but absolutely imperative if the interests of the insane are to be considered, and the cry raised a short time since for its total abolition was as silly as it well could be. We agree with Mr. Hill that a good subordinate staff is essential to careful treatment, and we further agree that "the greatest stimulant of all for long and efficient service—the certainty of a pension—surely ought not to be withheld." In turning over the leaves of asylum reports we have often been struck with the big balances which the farms show in their favour, and we have wished that our fortunes had led us to the land instead of to paper and ink. At Wandsworth we find a balance of £729 in favour of the farm, but on examining the balance-sheet we find nothing put down for rent of land, or for interest of capital. Neither is the method of valuation stated, nor the prices charged to the asylum, so that the table is useless for purposes of comparison. The weekly cost of maintenance is 9s. 6¼d.

### SURREY COUNTY ASYLUM AT BROOKWOOD.

One thousand and sixty-three patients were in residence on the last day of December, 1890, being an increase of 13 on the numbers for the previous year. The admissions during the year amount to 394, and the recoveries were 94. The percentage of recoveries on the admissions is given as 44·17, but this must be arrived at by excluding transfers. There were 83 deaths, giving a percentage on the admissions of 7·78. Of the year's admissions, 39 were driven insane by drink, and 81 by hereditary tendency. Every asylum report discloses similar facts, and yet drinking and imprudent marriages are as common as ever. Alas, that we have to write it! The following paragraph from Dr. Barton's report will find an echo in the breast of every unfortunate medical superintendent: "By the new Lunacy Act, which came into operation on May 1st, I find my responsibility very considerably increased. Much extra clerical work is thrust on the medical superintendent, who, being the person solely responsible by the statute that all the provisions of the Act, as regards the asylum, are strictly and properly carried out, is, in default, surrounded by penalties." Some of these provisions which Mr. Barton refers to could hardly be considered necessary when applied to private asylums, but why county asylums were saddled with them is known only to the framer of the Act. It is well known that many of our best Acts of Parliament have been those which repealed other Acts, and we must live in hope that the repeal of much of the Lunacy Act is not far distant. The interest of everyone connected with county asylums is to get rid of the patients and not to keep them, and it would not be difficult to show that the discharges are far too numerous. It is to be hoped the next Lunacy Act will recognise this. We do not find any farm account. The number of superior officials seems very high. There is a steward (with £250 a-year, while one of the medical officers has only £150), a clerk and two assistant clerks, a matron and two assistant matrons, a housekeeper, a dispenser, a head male attendant and a deputy head attendant. The weekly charge to the unions was 9s. 6d. per patient.

### THE HOLLOWAY SANATORIUM, VIRGINIA WATER.

When the year 1890 ended there were in this hospital 305 patients, being an increase of 63 during the year. Not fewer than 193 patients were admitted; 63 were discharged recovered, and 26 died. The percentage of recoveries would be 40·6, and the deaths 9 2. In 15 of the admissions drink was the cause of insanity, and hereditary influence in 56. Dr. Philipps' report shows evidence of much energy. A villa has been purchased in Brighton. It is intended as a convalescent home and as a place where patients can be sent for change of air and scene. At Virginia Water a cricket pavilion has been put up; new staircases have been built; sanitary arrangements have been improved; a corridor has been made between the chapel and the main building, and it is now proposed to light the hospital with electricity. When speaking of the New Lunacy Act Dr. Philipps says, "It has not apparently improved the position of the patient, and its practical suggestion that the person who comes under its provisions is to be treated as accused of a crime instead of suffering from a disease, and that those who have charge of such person are opponents instead of friends, raise difficulties and suspicions which must in some cases tend to hinder if not prevent recovery. The Act would be equally useful and less costly in working if three-fourths of the useless reports and forms which are required to be forwarded to the Commissioners in Lunacy, or kept in the hospital, were abolished." It is three years since we ventured to urge in these columns the desirableness of appointing lady physicians in our lunatic asylums, and at last we have the gratification of seeing our recommendation adopted at this asylum. The influenza visited the asylum in the early part of the year, and it is recorded that the staff suffered out of all proportion to the patients, a fact which has been noted in many institutions. We hope the next report will show the payments made for the patients, and the average weekly cost per head.

### NOTTINGHAM BOROUGH ASYLUM.

On December 31st, 1890, there were 544 patients in the asylum, as against 402 at the close of the previous year. During 1890, 250 patients were admitted, 56 recovered, and 39 died. The recoveries stand at the high percentage of 42·7, and the deaths at 8·7, which is below the average. Phthisis was the cause of death in four cases only. The Visiting Committee of this Asylum wisely decided to build in excess of their present requirements, and they are now reaping the benefit of their forethought; 110 patients have been received from the London County Asylums, and a pretty little margin of profit will accrue from this arrangement. The farm account shows a balance of £420 in favour of the farm. No estimate of rent is included in the account; no charge is made for patients' labour; no interest on capital; and the prices charged to the asylum for materials supplied are not stated. We could not find any table showing the cost per head per week—a curious omission.

### LINCOLN COUNTY ASYLUM.

During the year the number of patients fell from 672 to 662. There were 168 admissions, 62 recoveries, and 90 deaths; the two latter showing respectively the percentages of 39· and 13·3. As causes of the disease we find that drink was the factor in 16 cases, and hereditary influence in 94; or, in other words, 110 of the 168 admissions were owing to preventible causes. Ten of the deaths were due to phthisis, 15 to general paralysis, six to Bright's disease, and two to dysentery. The asylum was crowded so much that 20 patients were transferred to the South Yorkshire Asylum, making a total of 59 Lincolnshire patients in that asylum, and incurring an increase of cost to the Lincolnshire patients of £650 a-year. The balance in favour of the farm stands at £509. The rent of the land is charged; but we do not notice the interest on capital invested. The milk is charged at one shilling a-gallon to the asylum. He will be a lucky farmer who can get so much. The weekly rate of maintenance is 8s. 1½d.

